# *In vivo* characterization of microglial engulfment of dying neurons in the zebrafish spinal cord

**DOI:** 10.3389/fncel.2015.00321

**Published:** 2015-08-31

**Authors:** Marco Morsch, Rowan Radford, Albert Lee, Emily K. Don, Andrew P. Badrock, Thomas E. Hall, Nicholas J. Cole, Roger Chung

**Affiliations:** ^1^Motor Neuron Disease Research Group, Faculty of Medicine and Health Sciences, Macquarie UniversitySydney, NSW, Australia; ^2^Faculty of Life Sciences, The University of ManchesterManchester, UK; ^3^Division of Cell Biology and Molecular Medicine, Institute for Molecular Bioscience, The University of QueenslandBrisbane, QLD, Australia

**Keywords:** zebrafish, apoptosis, necrosis, imaging, M1, M2, glia, neuron

## Abstract

Microglia are specialized phagocytes in the vertebrate central nervous system (CNS). As the resident immune cells of the CNS they play an important role in the removal of dying neurons during both development and in several neuronal pathologies. Microglia have been shown to prevent the diffusion of damaging degradation products of dying neurons by engulfment and ingestion. Here we describe a live imaging approach that uses UV laser ablation to selectively stress and kill spinal neurons and visualize the clearance of neuronal remnants by microglia in the zebrafish spinal cord. *In vivo* imaging confirmed the motile nature of microglia within the uninjured spinal cord. However, selective neuronal ablation triggered rapid activation of microglia, leading to phagocytic uptake of neuronal debris by microglia within 20–30 min. This process of microglial engulfment is highly dynamic, involving the extension of processes toward the lesion site and consequently the ingestion of the dying neuron. 3D rendering analysis of time-lapse recordings revealed the formation of phagosome-like structures in the activated microglia located at the site of neuronal ablation. This real-time representation of microglial phagocytosis in the living zebrafish spinal cord provides novel opportunities to study the mechanisms of microglia-mediated neuronal clearance.

## Introduction

Microglia are the resident macrophages of the CNS and play crucial roles in mediating immune-related functions (Barron, 1995; Hanisch and Kettenmann, 2007; Graeber and Streit, 2010; Ransohoff and Cardona, 2010). Microglia patrol the entire vertebrate nervous system, where they can detect the presence of apoptotic and damaged neurons, and consequently engulf these cells to minimize the spread of neuronal debris. This microglial activity requires fast-acting communication between the two cell types, such that microglia are primed for rapid response to a variety of stimuli (such as dying neurons). However, many of the fundamental mechanisms that regulate the detection of injured neurons and subsequent microglial activation during phagocytosis still remain elusive. Short-term microglial activity is generally accepted to serve a neuroprotective role, while chronic activation has been implicated as a potential pathogenic mechanism in neurodegenerative disorders (Block et al., 2007). *In vitro* studies over the last decade have established the morphological transformations that microglia undergo during injury and disease, characterized by the transformation from a ramified morphology to an ameboid appearance, in a process termed “microglial activation” (Hanisch and Kettenmann, 2007; Kettenmann et al., 2011; Michaelis et al., 2015). Accordingly, microglia are now established as key players in the CNS, with their activation and inflammatory profile a major hallmark of CNS injury and neurodegenerative diseases (Block et al., 2007).

### Importance of clearance of dying cells by microglia

With a turnover of billions of cells as part of the normal homeostasis each day, prompt and efficient clearance is necessary to prevent secondary necrosis of dying cells and immune responses to autoantigens (Henson, 2005; Nagata et al., 2010). Irrespective of whether these cells are generated as part of normal development, tissue maintenance, or as part of adult neurogenesis, a striking feature of this phagocytic activation of microglia is that it occurs in an immunologically silent manner. This is in contradiction to the classical paradigm of microglial activation in disease states, where microglial activation is associated with inflammatory behavior such as cytokine expression (Smith et al., 2012). Microglial clearance of dying neurons can be distinguished into four steps: (1) “Find me” signals that attract microglia toward dying cells (Peter et al., 2010); (2) the dying cells then promote their fate through “eat me” signals that can be recognized by specific receptors on the microglia (Gardai et al., 2006); (3) physical interaction through engulfment of the dying cell by microglia (Ravichandran and Lorenz, 2007); and (4) the phago-lysosomal digestion of the engulfed cellular debris (Kinchen et al., 2008; Kinchen and Ravichandran, 2008).

### Advantages of zebrafish for real-time imaging of microglia function

*In vitro* studies have contributed extensively to improving our understanding of microglial function. However, certain questions can only be addressed using an *in vivo* system in which normal cell composition, morphology, and dynamics are preserved. This is particularly important when working with microglia, as these cells respond to even very small changes in the CNS, with their morphological properties often being used as readout of pathological conditions. Live-imaging in mice, with surgically thinned skulls, has opened up a new era of glial research as it has revealed for the first time that “resting” microglia are actually highly dynamic and use their long cellular extensions to scan the surrounding environment (Davalos et al., 2005; Nimmerjahn et al., 2005). More recently, the use of zebrafish for live-imaging studies of the nervous system has advanced the study of microglial activation toward elucidating specific molecular mechanisms involved in the engulfment of neurons (Peri and Nüsslein-Volhard, 2008; Sieger et al., 2012; Sieger and Peri, 2013; Mazaheri et al., 2014). Zebrafish offer unique advantages, including their small size and transparency (facilitating high resolution live-imaging microscopy), relatively easy and inexpensive maintenance, and ex-utero development (Westerfield, 2000). In accordance with their mammalian counterparts, zebrafish microglia are dynamic cells that form a non-overlapping network (with discrete domains) within the zebrafish spinal cord (Eyo and Dailey, 2013). Moreover, microglia, neurons and organelles of the microglial phagocytic pathway can be simultaneously labeled with spectrally distinct fluorophores, allowing live imaging of the entire microglial population in order to study the interaction between neurons and microglia (Li et al., 2012). Importantly, the molecular and cellular machineries of microglia to recognize and engulf dying neurons are conserved across vertebrates (Peri and Nüsslein-Volhard, 2008).

In this study, we have used time-lapse confocal microscopy to reveal that “resting” microglia in zebrafish are highly dynamic in the spinal cord in their non-activated form. In response to selective ablation of an individual spinal motor neuron, a single microglia migrates toward the site of injury within several minutes and subsequently ingests neuronal debris from the dying cell. Collectively, these studies provide new insight into the essential clearance of dying neurons in the spinal cord by microglia.

## Materials and methods

### Fish maintenance and transgenic lines

Zebrafish (*Danio rerio*) were maintained at 28°C in a 13 h light and 11 h dark cycle. Embryos were collected by natural spawning and raised at 28.5°C in E3 solution according to standard protocols (Westerfield, 2000). Experimental protocols were approved by Macquarie University Animal Ethics Committee (Zebrafish models of neural disorders; protocol no. 2012/050).

The behavior of “activated” microglia was studied between 48 hours post fertilization (hpf) and 5 days post fertilization (dpf). In order to allow high-resolution confocal live-imaging of individual neuron-microglia interactions, we utilized the following previously characterized zebrafish lines: *Tg(mpeg1:GAL4,UAS:mCherry) (gl22Tg)*, referred to as *mpeg1:mCherry* in the text (Ellett et al., 2011); *rwTg(isl1:GFP)*, referred to as *islet1:GFP* in the text (Higashijima et al., 2000); *Tg(met:GAL4,UAS:EGFP) (ed6)*, referred to as *cmet:GFP* in the text (Hall et al., 2007); s1020t*Et(-0.6hsp70l:GAL4-VP16) (s1020t) and Tg(UAS:Kaede)*, referred to as *s1020t:Kaede* in the text (Scott et al., 2007). Expression constructs and novel transgenic lines were generated using the Tol2 kit (Kwan et al., 2007). *Tg(mnx1:mKOFP2-CAAX) (mq7Tg)* (Flanagan-Steet et al., 2005; Arkhipova et al., 2012; Acosta et al., 2014), referred to as *mnx1:mKO2* in the text was generated using recombined p5E-mnx1 (-6 to -2869bp mnx1, Arkhipova et al., 2012), pME-mKO2caax (synthesised by GeneArt), p3E-pA (Kwan et al., 2007) and pDest-Tol2-pA2-acrys-EGFP (Berger and Currie, 2013). *Tg(-3.5ubb:secAnnexinV-mVenus) (mq8Tg)*, referred to as *ubiq:secAnnexinV-mVenus* in the text was generated using recombined p5E-ubb (Mosimann et al., 2011), pMEsecAnnexinA5-NS (Addgene ID 67718), p3E-mVenus (ID 67719) and pDEST-Tol2-pA2 (Kwan et al., 2007). pME-secAnnexinA5-NS was based on the initial design of Van Ham et al. (2010, 2012). It incorporates the human ANXA5 fused to a mammalian codon optimized consensus secretion signal. The *ubiq:secAnnexinV-mVenus* fish line expresses fluorescent AnnexinV ubiquitously throughout the zebrafish and allows the detection of any cell that expresses phosphatidylserine (PS) on the outer leaflet of the plasma membrane. PS is normally constrained to the inner leaflet of the plasma membrane and gets exposed to the outer leaflet in various conditions, including oxidative stress and apoptosis (Kuan et al., 2000; Valencia and Morán, 2001).

### UV ablation

Targeted ablation of individual neurons was achieved using the 405 nm UV laser of the Leica SP5 confocal microscope. Through an initial z-stack the middle plane of the soma of the neuron of interest was determined and set for UV ablation. The internal Leica FRAP software was used to manually outline the ablation region of interest (ROI; generally a circle or ellipse covering 30–50% of the cell body). For ablation the laser power levels were set between 50–80% of the maximal 405 nm laser power and the “zoom-in” function of the FRAP software was applied to maximize dwell time and laser ablate precisely the outlined area. Dwell time of the laser for neuronal ablation was generally set in a range of ~60s to assure immediate ablation of the neuron (irreversible loss of fluorescence) and to trigger microglial response. For photoconversion and dose-dependency measurements laser power levels were set between 15 and 90% and dwell time between 10 and 60s.

### Microscopy and imaging

For live-imaging, fish 2–5 dpf were anesthetized in 0.01% tricaine (w/v) and embedded in 1.5% (w/v) low-melting point agarose in glass-bottomed dishes (Westerfield, 2000). When the agarose was cooled down to room temperature the embedded fish were covered with E3 plus 0.01% (w/v) tricaine. The experiments were terminated by adding tricaine solution (4g/L) to the dish. Time-lapse imaging was started approximately 45 min after embedding and was carried out on an upright Leica SP5 confocal microscope at room temperature (22–24°C) with 40 × (NA 0.8) and 63 × (NA 0.9) water objectives and the argon laser lines (458, 476, 488, 496, and 514 nm) or tunable white-light laser (470–670 nm) respectively. A sub-set of preparations was imaged to count and measure the movement of the “surveying” microglia using a 10 × (NA 0.3) water objective. Usually, z-stacks spanning 10–15 planes (approximately 10–30 μm) were imaged (2–5 min; depending on size) every 3–8 min and collapsed to maximum intensity projections using ImageJ or Fiji software (http://imagej.nih.gov/; http://fiji.sc/Fiji). Images were brightness and contrast adjusted for visualization and illustration. Videos were created using the ImageJ export function or the Imaris software (Bitplane Imaris, Switzerland) for rendered visualizations.

### Analysis

ImageJ or Fiji software was used to analyze microglia size, movement, and time-course. Maximum intensity projections (MIP) of the time-lapse recordings were used to measure the area and movement of microglia staining as described previously (Tse et al., 2014). Briefly, MIP were thresholded and the area determined using the “Analyse–Measure” function in ImageJ at relevant time points. To measure the movement of the microglia, a straight line was drawn between the center of the cell at the different time points and the distance measured and added. Average speed of microglia was calculated by dividing the maximum distance by the time taken to capture the relevant z-stacks. Statistical analysis was performed with GraphPad Prism software (GraphPad Software, CA, USA). Microglial sizes before and after ablation were taken from the same cells and differences between the means were evaluated using a paired *t*-test. Microglia speed was averaged from microglia surveying the spinal cord and activated after ablation. Differences in speed, volume, and surface area were evaluated using an unpaired *t*-test. For all statistical tests significance was taken as *P* < 0.05. Unless otherwise indicated the data were symmetrically distributed with equivalent variances and values are presented as the mean ± standard error of the mean (SEM). Numbers of fish used for analysis were designated as N and number of cells as n.

### Imaris rendering

3-dimensional visualization and quantification of macrophages was performed using Imaris v7.7.2 (Bitplane, Switzerland). Morphometrics of *mpeg1:mCherry* microglia were rendered and tracked over time using the automated surfaces function. Images were smoothed at a detail level of 0.4 μm and a threshold established by background subtraction (local contrast) of 1.5 μm. Surfaces below 10 voxels were filtered from the algorithm. For 4D surface tracking the autoregressive motion algorithm was employed (gap closed and a maximum distance of 50 μm). The same parameters were used for each quantified image set. Only cells fully within the Z volume were included for analysis.

## Results

### *In vivo* live-imaging of spinal motor neurons and microglia in zebrafish

**Figure 1:**
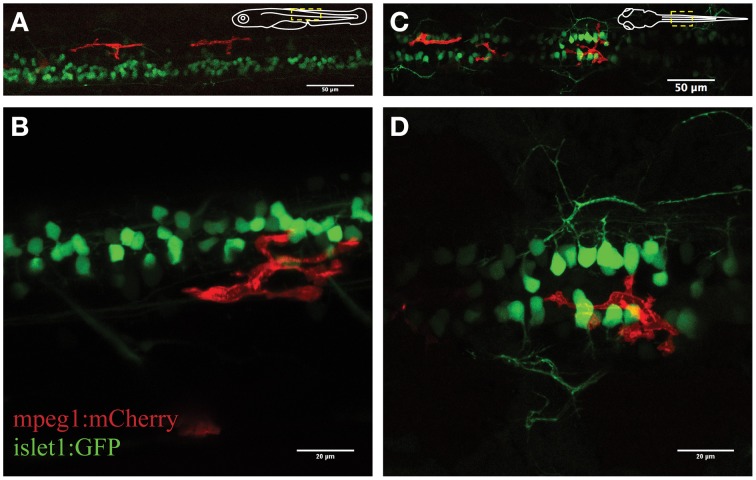
**Visualization of microglial activity in the spinal cord of 3 day old transgenic zebrafish expressing GFP-positive neurons (*islet1:GFP*) and mCherry-positive microglia (*mpeg1:mCherry*)**. **(A)** Overview (lateral) of the spinal cord and **(B)** enlarged lateral view of spinal neurons and a single microglia. **(C)** Dorsal view of the spinal cord microglia and **(D)** enlarged dorsal view. Schematic inserts in **(A,C)** depict orientation of the fish and outline the presented area.

We firstly characterized the behavior of microglia in the uninjured spinal cord in *mpeg1:mCherry* zebrafish. This line was chosen as the Gal4 regulatory element causes mosaic expression of mCherry in microglia, allowing us to confidently visualize individual microglia. Accordingly, we observed a non-overlapping distribution of fluorescent microglia throughout the spinal cord (on average 12 microglia ± 1.4; *N* = 7). The mpeg1-fluorescent cells were on average 240 μm^2^ (±15.4 μm^2^; *n* = 27; *N* = 21) in size and displayed several features that are characteristic of microglia in the zebrafish and mouse brain (Nimmerjahn et al., 2005; Ellett et al., 2011; Svahn et al., 2013). Hence, spinal microglia exhibited a branched morphology, with filopodia-like processes and bulbous-tipped processes that extended and retracted over minutes (Figure 1). A subset of microglial cells (~30%) showed a highly dynamic behavior, patrolling up and down the spinal cord. This behavior was obvious even in the absence of an “activating” trigger and without characteristic phagocytic activities, such as engulfment of neuronal structures or rapid extension/retraction of their processes. These motile microglia traveled at an average speed of 1.5 μm/min, averaging distances of 98 μm per hour (±7.7 μm; *n* = 18; *N* = 7) and a maximum of 241 μm within less than 2 h.

### Selective ablation of individual motor neurons within the spinal cord

**Figure 2:**
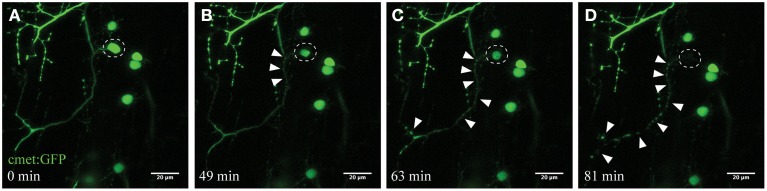
**Time-course imaging of the neurodegeneration of UV-ablated spinal neurons**. **(A–D)** UV-irradiation of a single spinal neuron (*cmet*:*GFP*; **A**; circle) resulted in the soma of the neuron shrinking over time, followed by axonal fragmentation (**B**; arrowheads). This axonal degeneration radiated anterogradely toward the distal end of the axon **(C)**, until finally the fluorescence in the soma disappears and the entire axon shows “blebbing” **(D)**. Scale bars = 20 μm. Supplementary Video 1 shows the time-lapse video of this process.

Microglia constantly and efficiently scan for any alterations in their microenvironment, ranging from changes in neuronal activity to signals of damage-associated processes (Nimmerjahn et al., 2005; Ransohoff and Perry, 2009). We applied UV laser ablation (405 nm) to the soma of spinal motor neurons to selectively induce a localized microglial response in the living zebrafish spinal cord. UV ablation of the fluorescent-labeled neurons (*cmet:GFP*) reproducibly led to selective death of the targeted neuron within minutes to hours (Figure 2). The time-course of neuronal death was dependent upon parameters such as laser-power, ablation size and dwell time. Ablated and dying neurons showed characteristic morphological changes, including the shrinkage of the cell soma with intact membrane structures (Figures 2A,B), and progressive anterograde degeneration (blebbing) commencing at the targeted soma and continuing along the axon over time (Figures 2C–D; Supplementary Video 1).

**Figure 3:**
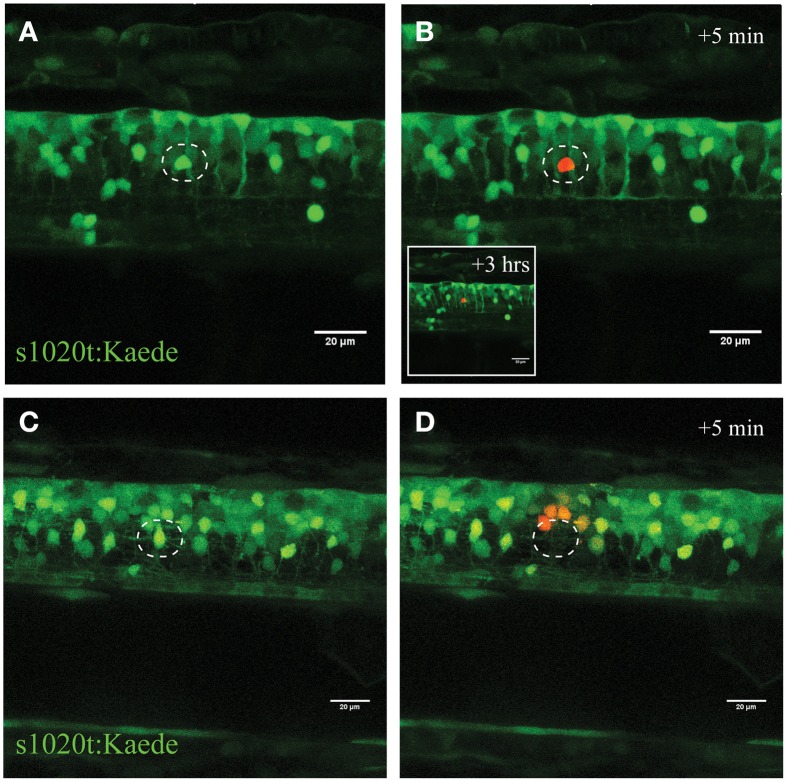
**Confirmation of single-cell UV irradiation through the activation of photoconvertable Kaede inside a motor neuron**. **(A–D)** UV irradiation of neurons labeled with the photoconvertable fluorophore Kaede. **(A)** Photoconversion of a single neuron (circle) with a laser power of 30% for 10 s led to photoconversion of Kaede (from green to red) in only the targeted individual neuron (**B**; insert illustrates the intact neuron 3 h post conversion). Ablation of a single neuron (**C**; circle) with a higher laser power (95% for 10 s) resulted in immediate disappearance of that neuron **(D)**, and subsequent photoconversion of Kaede in a small number of surrounding neurons in a radius of approximately 20 μm. Scale bars = 20 μm.

To validate this process of neuronal ablation and the specificity of this UV laser ablation method, we used transgenic zebrafish expressing the Kaede fluorophore in spinal neurons (*s1020t:Kaede*; Scott et al., 2007). Kaede is a photo convertible fluorophore that changes fluorescence from green to red after exposure to UV light (Ando et al., 2002). UV laser targeting the soma of an individual neuron with different laser power levels revealed the tuneable nature of this approach for selectively inducing immediate or delayed death of individual neurons. Accordingly, ablation with a laser power of 30% for 10 s led to the efficient photo-conversion of only a single green Kaede neuron amongst a dense cluster of other Kaede-labeled motor neurons in the spinal cord (Figures 3A,B). Importantly, this laser power resulted in no cell death of the targeted or surrounding neurons within the next 2 h. On the other hand, using high laser power at a near maximum of 95%, resulted in immediate ablation of the targeted neuron (Figures 3C,D white circle) and collateral photo conversion of surrounding neurons within a radius of approximately 20 μm (Figure 3D). Even though these surrounding neurons were exposed to some UV irradiation, we never observed any unspecific death within the direct proximity of the laser ablation site. Increasing the duration (longer dwell time) of the UV laser ablation with different laser power did not result in further spread of the photoconversion and was maintained in a radial diameter of approximately 25 μm (Supplementary Figure S1).

In summary, our high resolution time-lapse imaging laser ablation demonstrated neuronal blebbing, observed as the development of bead-like formations along the axon and granular disintegration distal to the site of UV-induced injury, and ultimately death of the neuron (Figure 2). Reducing the laser power further provided an opportunity to stress the neuron without inducing death (Figures 3A,B), whereas raising the laser power to near maximal levels resulted in the immediate death of the targeted neuron with minimal effect on the surrounding cells (Figures 3C,D). Consequently, this approach of targeting neurons with varying intensities of UV light represents a novel and reliable method to selectively induce stress or death to individual spinal (motor) neurons in the living zebrafish.

### Dying motor neurons express apoptotic markers associated with phagocytic signaling

**Figure 4:**
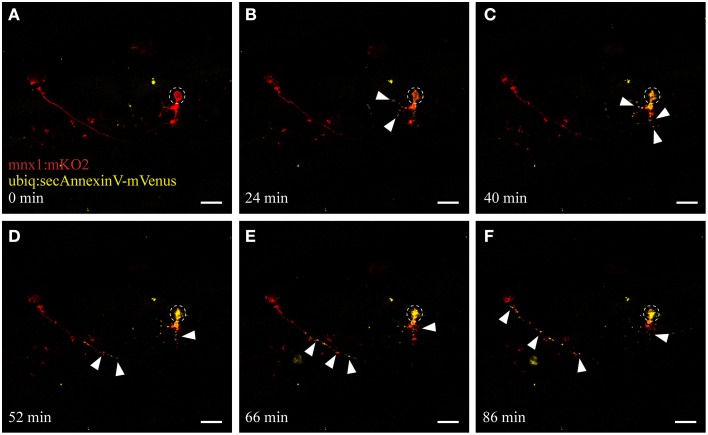
**UV ablation activates annexinV-mediated neuronal apoptosis**. UV ablation of a single neuron in a transgenic fish expressing the neuronal marker *mnx1:mKO2* as well as the apoptotic marker AnnexinV (*ubiq:secAnnexinV-mVenus*). Shortly, after UV ablation of the soma of the neuron (**A**; circle), phosphatidylserine (PS) gets switched to the outer leaflet of the plasma membrane and trapped by the AnnexinV marker indicated by the yellow fluorescence (**B,C**, arrowheads). Throughout the time-course of neuronal degeneration, AnnexinV puncta (yellow fluorescence) were present within the degenerating cell body **(C,D)** as well as along the axon, progressing anterogradely from the site of ablation **(D–F)**. Scale bars = 20 μm. Supplementary Video 2 shows the time-lapse video of this process.

When cells undergo stress or death, they release “eat-me” signals that mediate the rapid recognition and engulfment of these dying neurons and neuronal debris to avoid a spread of inflammation. Eat-me signals, such as the phospholipid phosphatidylserine (PS) act as crucial detection signals for the recognition and ultimately the efficient digestion of these cells (Davalos et al., 2005; Takahashi et al., 2005; Ravichandran, 2011; Brown and Neher, 2014). We utilized AnnexinV, a protein that binds to PS lipids exposed on apoptotic cells (Vermes et al., 1995), to visualize this apoptotic/phagocytic signaling process *in vivo*. We generated a stable transgenic line (*ubiq:secAnnexinV-mVenus*; see Materials and Methods) and crossed these fish with the *mnx1:mKO2* motor neuron line to selectively use this reporter to detect dying neurons in the spinal cord. UV ablation of motor neurons in these double-labeled fish showed the same consistent morphological changes of somal degeneration and axonal blebbing as described earlier (compare Figure 4 and Figure 2; Supplementary Video 2). Moreover, within several minutes after ablation we detected the activation and accumulation of AnnexinV at the neuron (Figure 4B, yellow channel), firstly at the targeted soma site and then progressively along the axon toward the more distal parts of the neuron. As the neuron degenerated, AnnexinV-labeled fragments of neuronal debris were observed around the ablation site (Figures 4D–F).

### Activated microglia engulf and accumulate neuronal debris

**Figure 5:**
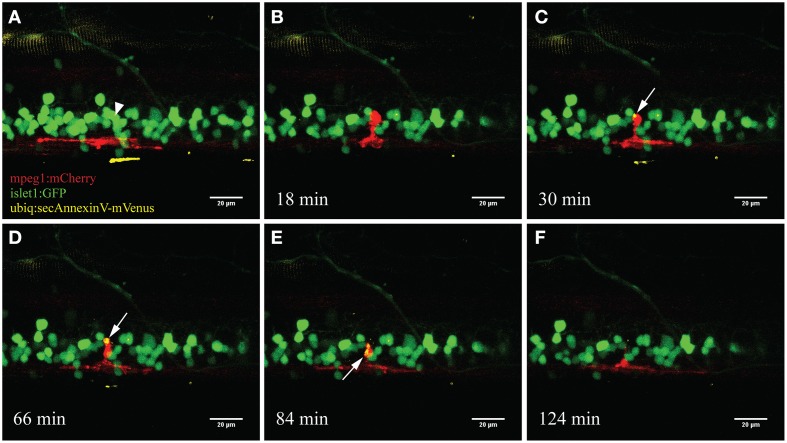
**Accumulation of the apoptotic marker AnnexinV within microglia**. Upon ablation of a neuron in the spinal cord (**A**; arrowhead) the surrounding microglia moved toward the ablation site **(B)**. After several minutes the fluorescently labeled marker AnnexinV lights up within the microglia (**C**; arrow). AnnexinV accumulation within the microglia increases over time **(D)**, till the phagocytic debris gets transported away from the ablation site **(E)** and conceivably degraded resulting in disappearance of the fluorescence (**F**). Scale bars = 20 μm. Supplementary Video 3 shows the time-lapse video of this process.

To specifically visualize the engulfment of neuronal remnants from dying neurons, we used a triple labeled fish (*islet1:GFP; mpeg1:mCherry; ubiq:secAnnexinV-mVenus*) to visualize this process following our UV ablation approach. AnnexinV-mVenus accumulated in the phagocytic vesicles of the activated microglia after neuronal ablation (Figure 5). Over a time frame of approximately 2 hours, AnnexinV accumulated within the cytoplasm of the microglial cell and subsequently was transported away from the ablation site.

### Microglia rapidly respond to, and phagocytose dying spinal motor neurons

**Figure 6:**
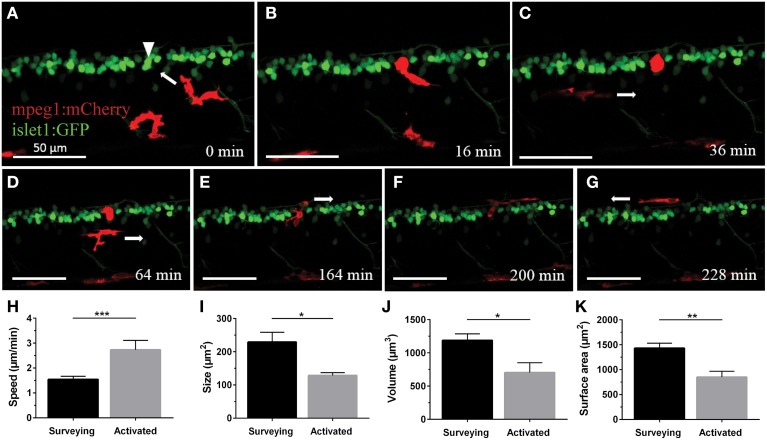
**Microglia rapidly respond and are recruited to the site of neuronal ablation**. UV ablation of a GFP-expressing spinal neuron (**A**; arrowhead) resulted in only one of two surrounding microglia (red) relocating and phagocytosing the dying neuron **(B,C)**. Over a period of several hours, other microglia pass by the phagocytosing microglia **(D)**, and after approximately 2.5 h the phagocytosing glial cell changes morphology back to a stellate morphology **(E)**, indicating termination of the phagocytosis process **(F,G)**. Supplementary Video 4 shows the time-lapse video of this process. Scale bars = 50 μm. **(H–K)** Quantitative analysis of morphometric changes in microglial response to dying spinal neurons. Microglia undergo significant changes in morphology **(I–K)** and speed **(H)** upon activation. ^*^p < 0.05 paired Student's *t*-test; *n* = 6–7; *N* = 6–7; 2D ImageJ analysis **(H)**; ^***^p < 0.001 unpaired Student's *t*-test; *n* = 7–22; *N* = 6–9; 2D ImageJ analysis **(I)**; ^*^p < 0.05; ^**^p < 0.01 unpaired Student's *t*-test; *n* = 4–13; *N* = 4–10; 3D Imaris analysis **(J,K)**.

In order to characterize the phagocytosis of dying neurons via microglial engulfment in the spinal cord, we generated transgenic lines in which zebrafish expressed together green fluorescent neurons (*islet1:GFP*) and a subset of red microglia (*mpeg1:mCherry*; Figures 1, 5, 6). The microglial population in these fish showed a ramified morphology that was reminiscent of reports from the (fish) brain (Peri and Nüsslein-Volhard, 2008; Svahn et al., 2013). Consistent with our *mpeg1* fish line that expressed fluorescence in a subset of microglia (Gal4:UAS), UV laser ablation of some neurons did not lead to a response of the fluorescent microglia. In approximately 60% of our ablations we did not observe subsequent microglia engulfment, conceivably because non-fluorescent microglia would have responded to ensure the immediate uptake of the neuronal debris. In successful experiments, time-lapse imaging revealed that soon after UV laser ablation of a single motor neuron, individual microglia underwent dramatic changes in morphology by extending and retracting processes, moving toward the site of the ablated neuron and changing to a spherical shape within minutes (Figure 6). The typical first response was for microglia to extend phagocytic protrusions toward the ablated neuron body, therefore shifting the whole microglia body toward the lesion site. This process took on average 27 min until complete engulfment of the ablated soma was achieved (Table 1). Notably, this process was characterized by a remarkable increase in the dynamic behavior of the microglia as it moved toward the dying neuron (Supplementary Video 4). Within minutes, activated microglia doubled their speed (2.7 μm/min vs. 1.5 μm/min in the “surveying” state; Table 1; Figures 6H–K), covered substantial distances of 33–94 μm toward the lesion site (72.8 μm on average), and decreased in size significantly to form a round amoeboid body as it engulfed the neuron remnants (128.9 μm^2^ vs. 229.4 μm^2^ before activation; Table 1; Figures 6H–K).

**Table 1:** **Morphometric characteristics of microglia before and after activation in response to UV-ablated spinal neurons**.

	**“Surveying state”**				**“Activated state”**				
		**SEM**	**n=**	**N=**		**SEM**	**n=**	**N=**	**P**
**ImageJ ANALYSIS**									
Average size microglia in spinal cord (μm^2^)	244.4	±15.4	27	21					
Average number of microglia (n)	12	±1.4		7					
Average distance moved per hour (μm)	97.8	±7.7	18	7					
Average size (μm^2^)	229.4	±29.3	7	7	128.9	±8.2	6	6	^*^^a^
Average speed (μm/min)	1.5	±0.1	22	7	2.7	±0.3	7	7	^***^^b^
Average time to engulf (min)					27.3	±1.9	7	7	
Average distance moved toward ablation site (μm)					72.8	±8.7	7	7	
**IMARIS ANALYSIS**									
Average volume microglia (μm^3^)	1189.5	±97.9	13	10	703.8	±148.0	4	4	^*^^b^
Surface area (μm^2^)	1433.3	±98.0	13	10	852.6	±115.9	4	4	^**^^b^
Average speed (μm/min)	1.4	±0.27	4	4	2.5	±0.2	4	4	^*^^b^
Distance moved (from start to area ablated, μm)					72.3	±7.5	4	4	

Numbers of fish were designated as N and number of cells as n. P-values indicate statistical significance (

a paired t-test;

b unpaired t-test).

* p < 0.05;

** p < 0.01;

*** *p < 0.001*.

Interestingly, occasionally multiple microglia showed clear signs of activation by rushing toward the lesion site. However, in all cases only a single fluorescent microglia (the first) remained at the site of injury to clear the neuronal debris. Over the period of several hours during which microglial clearance took place (on average 251 min until the microglia moved away again; ±34.6 min; *n* = 4; *N* = 4), other microglia would inspect the site in close proximity without interfering with the ongoing clearance, seemingly as if they were aware of the ongoing phagocytosis by another microglia (Supplementary Video 4). The mechanism(s) governing this selective uptake of a dying neuron by a single macrophage in the presence of activation signals remain unknown. Microglia that were clearing a lesion site remained in their activated spherical shape for up to 2 h (average of 86 min; ±22.6 min; *n* = 4; *N* = 4). Upon completion of clearance the microglial cell transitioned back to a branched morphology by extending processes forth and back again, and migrating away from the ablation site (Figures 6E–G; Supplementary Video 4).

### 3D modeling of phagocytosing microglia

**Figure 7:**
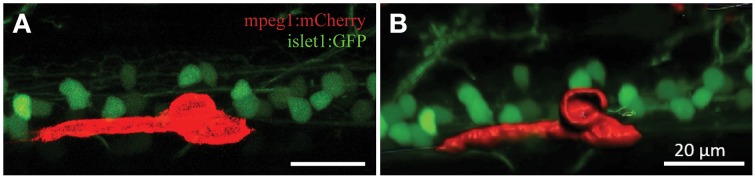
**3D rendering demonstrates dynamic morphological features of activated microglia**. Confocal live-imaging alone provides basic morphometric information of microglia phagocytosing an UV-ablated spinal neuron **(A)**. Imaris 3D rendering of this same single microglia revealed the formation of a phagosome-like structure **(B)**, which presumably facilitates engulfment of the neuronal remnants. Supplementary Videos 5, 6 show the time-lapse videos of this process for comparison. Scale bars = 20 μm.

Confocal live-imaging alone provides only limited morphometric information upon phagocytosing microglia. Using three-dimensional rendering of time-lapse responses (Imaris), we found repeatedly that phagocytosing microglial cells extend bulbous-like protrusions tipped with phagocytic cups (Figure 7). For example, we observed a single microglia engulfing the neuronal remnants of an UV-ablated spinal neuron through formation of a phagosome-like structure (10 μm in size; Figure 7B, Supplementary Video 6) that cannot readily be resolved through standard confocal microscopy (Figure 7A; Supplementary Video 5). Importantly, this live-imaging observation is in line with scanning electron microscopy data showing that indeed phagosomes form a tight fitting around apoptotic particles (Krysko et al., 2003, 2006). Collectively, our data strongly demonstrates that microglia (in the zebrafish spinal cord) form bulbous-like phagocytic cups to engulf neuronal remnants, equivalent to the phagocytic behavior of mammalian microglia in the brain. Morphometric rendering analysis of a subset of these microglial cells with Imaris confirmed the speed and distances reported above (Table 1). It furthermore revealed that these microglial cells condensed in size during activation, demonstrated by a ~40% reduction in their average volume and surface area (1189.5 μm^3^ vs. 703.8 μm^3^; 1433.3 μm^2^ vs. 852.6 μm^2^; Table 1; Figures 6H–K).

## Discussion

We have studied the nature of microglial behavior through live-imaging approaches in the spinal cord of zebrafish. We found that microglia in the spinal cord are dynamic, constantly migrating through the spinal cord, seemingly surveying the environment for signs of disturbance. Accordingly, we show that microglia rapidly respond to the ablation of a single spinal neuron. We establish here that microglia immediately recognize dying neurons (most likely through “eat-me” signals like Phosphatidylserine and AnnexinV recognition) and effectively clear the neuronal debris by engulfing the lesion site. This single-cell resolution analysis combined with 3D rendering methods has uncovered that microglia efficiently ingest apoptotic material via the formation of cup-shaped phagosome-like structures.

Previous work has shown that Mpeg1 is expressed by the early myeloid originating macrophages that colonize the CNS throughout development (Herbomel et al., 2001; Ellett et al., 2011; Svahn et al., 2013). Svahn and colleagues have shown that the Mpeg1-labeled cells are the same macrophages that are under control of the *apoE* promoter and that have been observed in previous zebrafish live-imaging studies (Herbomel et al., 2001; Peri and Nüsslein-Volhard, 2008; Svahn et al., 2013). In our analysis, we observed that a subset of microglia from day 2 onwards actively survey the spinal cord by covering substantial distances (98 μm on average per hour). While we cannot exclude that temperature and/or anesthetics had an influence on microglial motility and function, other *in vivo* studies in mice and zebrafish have also reported a highly dynamic nature of microglia branching with speeds similar to what we have shown here (1.47 μm/min, Nimmerjahn et al., 2005; 2.5 μm/min, Peri and Nüsslein-Volhard, 2008; 0.3 μm/min, Svahn et al., 2013).

To study microglial responses to dying neurons in real-time, we used a UV laser ablation technique to induce targeted neuronal injury. Photoconversion experiments of the Kaede fluorophore revealed that this method induces dose-dependent cell death with little scattering to the surrounding tissue (Figures 2, 3; Supplementary Figure S1). Different types of laser have been used in previous zebrafish studies to induce injury to the kidney, heart, and brain as well as prompting thrombosis (Jagadeeswaran et al., 2006; Johnson et al., 2011; Sieger et al., 2012; Matrone et al., 2013). In Drosophila and epithelial cells, Soustelle and colleagues applied a very similar UV ablation approach to the one described here (Soustelle et al., 2008). However, we are not aware of any studies that have selectively demonstrated single-cell stress/death through UV-irradiation in the zebrafish spinal cord. While most studies have not characterized the type of (programmed) cell death they elicited through laser ablation, Matrone and colleagues reported histological changes suggestive of necrosis and apoptosis following localized laser injury of the heart (Matrone et al., 2013). Other studies demonstrated increased caspase activity and TUNEL-staining in the developing zebrafish embryo after severe stress conditions such as UV irradiation (Yabu et al., 2001). Accordingly, our studies using single-cell resolution imaging of spinal motor neurons confirmed the anterograde degeneration of the UV targeted neurons, and revealed rapid recruitment of AnnexinV, a marker of phosphatidylserine-positive apoptotic cells within minutes after ablation (Figure 4; Supplementary Video 2). Throughout the next hours the formation of PS-positive fragments became obvious along the axonal projections. Equally, cellular blebbing (fragmentation of cellular bodies) at the soma and the axons occurred consistently in our experiments when we stressed the neuron with intermediate laser power. Based upon these parameters, we demonstrate that the programmed cell death that we have triggered with our UV ablation approach has characteristic features of apoptotic cell death.

Microglia undergo complex interactions with other glial cells and neurons upon stress or injury. Most studies have focused upon microglial behavior in the brain during development or after injury. In our work, we have focused on the spinal cord of the zebrafish and have developed a robust method to study the behavior of microglia under normal physiological conditions. We show that after UV ablation the apoptotic marker AnnexinV accumulates in the phagocytic vesicles of the activated microglia (Figure 5). Importantly, when we selectively trigger microglial activation to visualize the immediate response to neuronal death, we show that microglia increase their speed significantly upon detection of neuronal injury, doubling their speed for a short period of time to reach the site of injury (Figure 6; Table 1). Since our approach allows the observation of microglial behavior before and after manipulation in the same experiment, we propose that this acceleration in movement is a clear example of specific activation of this microglia. It is important to note that our studies utilized an approach incorporating mosaic fluorophore labeling of microglia. Accordingly, in about 60% of imaging studies we did not observe a microglia phagocytose a neuron, presumably because the microglia was non-labeled. This indicates that the imaging itself did not activate the labeled microglia. While noting that there might be differences between microglial behavior in the brain vs. the spinal cord, our data are in accordance with a previous study in zebrafish brain which reported microglial migration rates of 2.5–3.5 μm/min toward apoptotic cells (Mazaheri et al., 2014).

An important advancement of our study is the ability to observe the response of individual microglia to the degeneration of a single spinal motor neuron. Our experiments consistently reveal that it is always only one microglia that ultimately phagocytoses the neuronal debris of the dying neuron. Indeed, following ablation of a single spinal neuron we have observed rapid recruitment of a single microglia, yet other microglia rush toward the lesion site, stop or slow down in close proximity but then pass by, seemingly as if they know that the degenerating neuron is already engulfed by another glial cell (Figure 6; Supplementary Video 4). This behavior is a level of complexity above the chemotactic diffusion model of attraction that has been reported previously, where injections of a bolus of ATP into the brain attract multiple microglia to the site of injection (Davalos et al., 2005; Sieger et al., 2012). Our studies suggest a higher order of regulation, such that microglia can sense when they are required to respond to a dying neuron or when no response is required. It will be interesting to differentiate the different molecular profiles of “resting” and “activated” glial cells in more detail in future studies.

The morphometric characteristics of microglial phagocytosis have primarily been identified through histological approaches. Although other studies have previously reported the (two-dimensional) formation of phagosome-like structures during microglial ingestion (Peri and Nüsslein-Volhard, 2008), the three-dimensional visualization of these phagosomes has, to the best of our knowledge, not been demonstrated in live-imaging studies. Precise visualization of the process of internalization of apoptotic bodies by macrophages was limited to scanning electron microscopy studies (Krysko et al., 2006). By applying 3D rendering techniques we consistently observed the formation of tight-fitting phagosome-like structure around the soma of dying neurons (Figure 7; Supplementary Video 6). Thus, this method provides a significant advancement in the visualization of live-imaging microglial phagocytosis to unambiguously demonstrate the formation of a phagosomal cup-shaped structure to capture neuronal fragments.

Combination of the visualization and ablation techniques with markers of inflammation in future studies will further advance our knowledge of microglia activation and homeostasis. Understanding these processes is critical as microglial clearance is symptomatic for many neurodegenerative diseases including motor neuron disease, where glial activation has been shown to contribute to the death of motor neurons (Philips and Robberecht, 2011).

## Author contributions

Conceived and designed the experiments: MM and RC. Performed the experiments: MM and RR. Analyzed the data: MM and RR. Contributed reagents/materials/analysis tools: MM, RR, RC, AL, ED, AB, TH, and NC. Provided technical assistance: MM, RR, RC, AL, ED, AB, TH, and NC. Wrote the paper: MM and RC. All authors contributed to revision of the article, and approved the final version of the manuscript.

### Conflict of interest statement

The authors declare that the research was conducted in the absence of any commercial or financial relationships that could be construed as a potential conflict of interest.

## Supplementary material

Supplementary Video 1 **A GFP-expressing spinal motor neuron is UV ablated, resulting in the rapid loss of GFP from the soma and blebbing of the axonal remnants (video time course is 90 min, 5 min per frame)**.

Supplementary Video 2 **A single spinal motor neuron (red) was targeted by UV laser ablation**. The ubiquitously expressed apoptotic marker AnnexinV (yellow) appears at the ablation site within ~12 min, indicating induction of apoptotic-like cell death in this spinal neuron (4 min/frame, overall duration ~2h).

Supplementary Video 3 **After UV ablation of a spinal neuron (green) a fluorescently labeled microglia (red) gets activated to take up the neuronal debris via phagocytosis**. Our apoptotic reporter fish *ubiq:secAnnexinV-mVenus* shows consequently the accumulation and transport of the fluorescently labeled apoptotic debris (yellow) within the microglia.

Supplementary Video 4 **UV ablation of a GFP-expressing spinal cord motor neuron (green) results in immediate microglia (red) response**. One of the two surrounding microglia quickly relocates toward the ablated neuron and starts to phagocytose the remnants of the dying neuron. This activated microglia displays a profound change in morphology (from a ramified appearance to a spherical shape). After approximately 2.5 h the phagocytosing glial cell reverts back to a ramified morphology, indicating termination of the phagocytosis process. Interestingly only one microglia actively phagocytoses the dying neuron while other microglia pass by and ignore the site of neuronal ablation, even after the phagocytosing microglia has departed (6 min/frame, overall duration ~10 h).

Supplementary Video 5 **UV ablation of a single spinal cord motor neuron (green) triggers an immediate microglia (red) response**. A single microglia migrates towards the ablation site and phagocytoses the neural remnants (5 min/frame, overall duration ~3.5 h). The 3D rendered version can be found at Supplementary Video 6.

Supplementary Video 6 **Imaris 3D-rendering of the same microglia response shown at Supplementary Video 5**. This method clearly reveals the formation of a phagosome-like structure (5 min/frame).

Supplementary Figure S1 **UV laser irradiation induces neuronal damage in a dose-dependent manner**. UV laser ablation with different laser power (**A**: 15%; **B**: 30%; **C**: 60%, and **D**: 90%) of the s1020t:Kaede expressing neurons (green) in the spinal cord shows the focal spread of the UV laser, indicated by the photoconversion (red). Using a low laser power (**A**) results in focused Kaede photoconversion (from green to red fluorescence) limited to a single targeted neuron (upper row: lateral view; lower row: dorsal view). Higher laser power (**B–D**) results in radial diffusion of the UV laser of approximately 40–50 μm. All laser ablation sites were conducted using a circular region of interest of ~1.5 μm in diameter, focused upon an individual soma of a neuron, with a laser dwell time of ~60 seconds in each experiment. Scale bar = 20μm.
