# Supplementary figures and images for: In vivo characterization of microglial engulfment of dying neurons in the zebrafish spinal cord
Source: Front Cell Neurosci. 2015 Aug 31;9:321. doi: 10.3389/fncel.2015.00321 (PMC4553390; doi:10.3389/fncel.2015.00321)

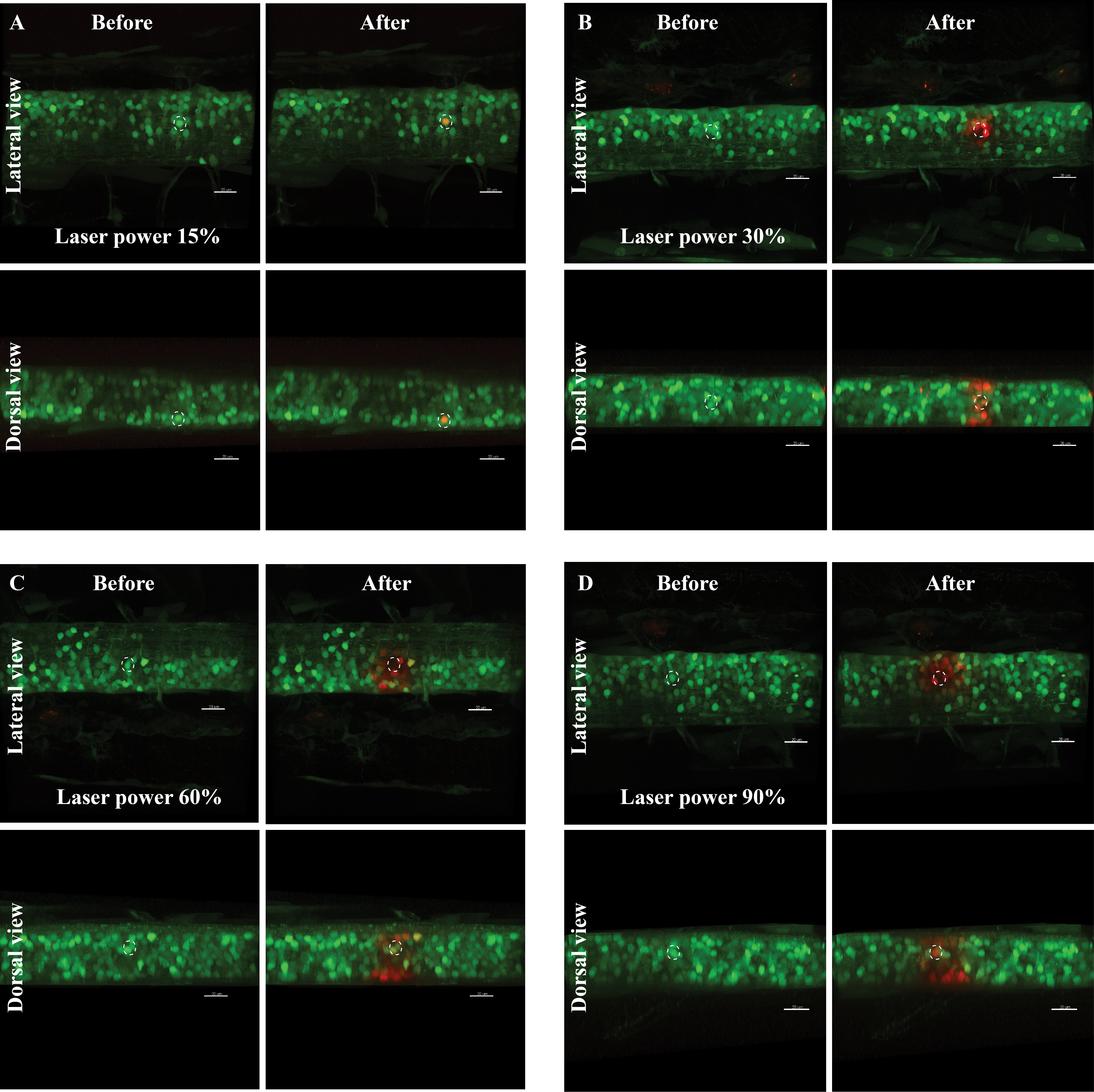

Supplement: Supplementary Figure S1 — UV laser irradiation induces neuronal damage in a dose-dependent manner. UV laser ablation with different laser power (A: 15%; B: 30%; C: 60%, and D: 90%) of the s1020t:Kaede expressing neurons (green) in the spinal cord shows the focal spread of the UV laser, indicated by the photoconversion (red). Using a low laser power (A) results in focused Kaede photoconversion (from green to red fluorescence) limited to a single targeted neuron (upper row: lateral view; lower row: dorsal view). Higher laser power (B–D) results in radial diffusion of the UV laser of approximately 40–50 μm. All laser ablation sites were conducted using a circular region of interest of ~1.5 μm in diameter, focused upon an individual soma of a neuron, with a laser dwell time of ~60 seconds in each experiment. Scale bar = 20μm. [file Image1.JPEG]
